# Single and Repeated Episodes of *Candida* Species Isolated From Cerebrospinal Fluid for Diagnosing Probable *Candida meningitis*

**DOI:** 10.3389/fmicb.2021.742931

**Published:** 2021-10-15

**Authors:** Lijun Xu, Handan Zhao, Minghan Zhou, Guanjing Lang, Haiyan Lou

**Affiliations:** ^1^National Clinical Research Center for Infectious Diseases, The First Affiliated Hospital, College of Medicine, Zhejiang University, Hangzhou, China; ^2^The State Key Laboratory for Diagnosis and Treatment of Infectious Diseases, The First Affiliated Hospital, College of Medicine, Zhejiang University, Hangzhou, China; ^3^College of Medicine, Zhejiang University, Hangzhou, China; ^4^Department of Radiology, The First Affiliated Hospital, College of Medicine, Zhejiang University, Hangzhou, China

**Keywords:** *Candida meningitis*, neurosurgery, cerebrospinal fluid, central nervous system, fungal infection, diagnosis

## Abstract

**Background:** The clinical relevance of single or repeated episodes of *Candida* spp. in cerebrospinal fluid (CSF) in adult patients is debatable.

**Methods:** Forty-two patients with positive *Candida* episodes in CSF were enrolled in this retrospective study.

**Results:** A total of 42.9% (18/42) were determined to have probable *Candida meningitis* (PCM). Neurosurgery [odds ratio (OR) (95% confidence interval), OR: 14.4 (1.6–126.1), *P* = 0.004], lumbar drainage [OR: 5.8 (1.5–23.3), *P* = 0.009], VP shunt [(OR: 5.6 (1.2–25.8), *P* = 0.020)], external ventricular drainage [OR: 4.7 (1.3–17.7), *P* = 0.018], CRP ≥ 10.0 mg/L [OR: 4.9 (1.3–18.1), *P* = 0.034], and postsurgical broad-spectrum antibiotics [OR: 9.5 (1.8–50.5), *P* = 0.004] were risk factors associated with PCM. A single CSF *Candida* episode for the diagnosis of PCM had 7.7% (0.4–37.9%) sensitivity and 20.7% (8.7–40.3%) specificity, whereas repeated episodes of *Candida* had 66.7% (41.2–85.6%) sensitivity and 95.8% (76.9–99.8%) specificity. No significant difference was found in radiological imaging or CSF profiles between PCM and non-PCM patients. A total of 37.5% (9/24) of patients without PCM received empirical antifungal treatment, and 88.9% (16/18) of patients with PCM received preemptive antifungal treatment. PCM patients had hospitalized mortality rates of 50.0% (9/18). The odds ratio of mortality was 23.0 (2.5–208.6) for PCM patients compared with non-PCM patients (*P* = 0.001).

**Conclusion:** Both single and repeated positive CSF samples have low validity for the diagnosis of PCM, suggesting that novel strategies for diagnosis algorithms of PCM are urgently needed. Empirical antifungal treatment should be started immediately for suspicious patients with risk factors.

## Introduction

*Candida meningitis* (*C. meningitis*) is rare, but severe cases have been encountered in clinical practice. A previous publication reported that *C. meningitis* mainly presented in neonates with extremely low birth weight due to blood brain barrier immaturity (Marr et al., 1994; Cohen-Wolkowiez et al., 2007; Agarwal et al., 2008; Ancalle et al., 2010). Central nervous system (CNS) *Candida* infections in adult patients are even rarer in clinical practice and are depicted only in some case reports. Those limited case reports suggest that CNS candidiasis is observed mainly in patients with predisposing conditions, such as hematological malignancy, organ transplant, intravenous drug use, diabetes mellitus, or human immunodeficiency virus (HIV) (Montero et al., 2000; Donnelly et al., 2020; Chaussade et al., 2021). Furthermore, neurosurgery history or foreign intracranial material are also risk factors for the development of CNS *Candida* infection (Chen et al., 2002; Fadel et al., 2018).

Although CNS *Candida* infection is a critical event in clinical practice, the proof and criteria of diagnosis for *C. meningitis* are ambiguous. Currently, the diagnosis of *C. meningitis* is dependent mainly on the culture of *Candida* spp. in cerebrospinal fluid (CSF). However, the relative importance of *Candida* spp. isolated from CSF by different routes remains unclear. For example, a study including 21 neurosurgical patients indicated that Candida species were isolated from multiple CSF samples in 10 cases and only isolated from CSF through indwelling devices in 11 cases. Interestingly, none of the 9 of 11 patients from whom *Candida* spp. were isolated from indwelling devices and who were not treated with antifungal regimens died of infection (Geers and Gordon, 1999). These data suggested that a single positive CSF sample drawn through an indwelling device is insufficient for a reliable diagnosis in patients receiving neurosurgery, and repeated positive CSF samples from drainage devices might be necessary for diagnosing *C. meningitis*. Furthermore, *Candida* cultures from CSF have poor sensitivity and can take a few days to grow. Unlike *Cryptococcus* infections, there is currently no widely accepted molecular-, antigen-, or nucleic acid-based testing method to expedite identification of the organism; thus, delaying the diagnosis and treatment of *C. meningitis* is of great importance in clinical practice.

The emerging occurrence of *C. meningitis* and the ambiguity of CNS *Candida* spp. diagnosis prompted us to conduct a retrospective study to summarize the utility of the different numbers and compositions of CSF *Candida* spp., CSF profiles and CT/MRI findings on the diagnosis of *C. meningitis* in patients with risk factors.

## Materials and Methods

### Study Cohort and Patient Enrollment

**FIGURE 1:**
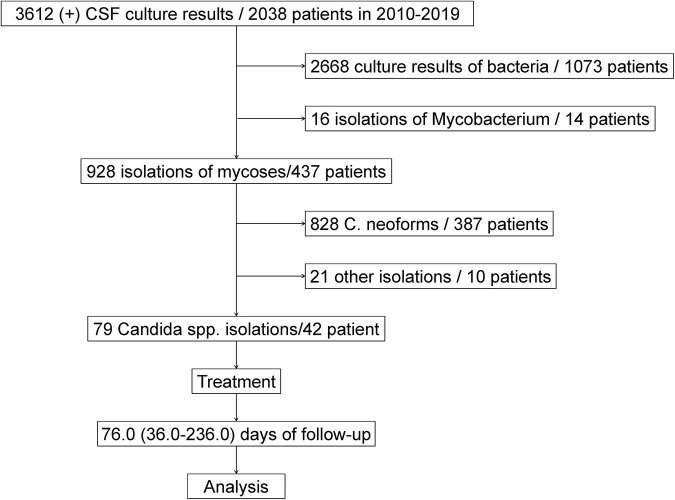
Flowchart of patient selection.

Between January 2010 and December 2019, a total of 3612 CSF-positive culture results were obtained from 2,038 patients from the First Affiliated Hospital, School of Medicine of Zhejiang University, Hangzhou, China. Of those, 2,668 culture results were bacteria from 1,073 patients, 16 isolations were mycobacterium or non-Mycobacterium infection from 14 patients, and 928 isolations were fungal infection from 437 patients. There were 387 patients with 828 *Cryptococcus* spp. isolations, 42 patients with 79 *Candida* spp. isolations and 10 patients with 21 other fungal infections in CSF culture (Zhao et al., 2021). Ultimately, the 42 patients from whom *Candida* spp. were isolated were enrolled in our cohort study. The flowchart of patient selection is briefly described in Figure 1.

### Diagnosis Criteria of Probable *Candida meningitis*

The diagnosis of probable *C. meningitis* (PCM) was made according to the following criteria: (1) at least one new onset of neurologic symptoms or signs such as fever, headache, vomiting/nausea, mental change, and encephalocele; (2) at least one episode of positive CSF *Candida* spp.; (3) a CSF profile that supported an infectious process, such as pleocytosis, elevated protein levels, and decreased glucose; and (4) the lack of an adequate treatment response to presumed bacterial or mycobacterial meningitis.

### Antifungal Susceptibility Testing

The broth dilution quantitative method was used to determine the minimum inhibitory concentration (MIC) of an antimicrobial agent that inhibited the growth of organisms *in vitro*. Antifungal analysis was automatically performed by the VITEK^®^ 2 COMPACT system and Etest package insert (Biomérieux, Marcy-l’Etoile, France).

### Follow-Up and Collection of Clinical Data

Basic medical information of patients (such as name, sex, BMI, blood test, imaging examination, and treatments) was recorded and saved in the electronic medical records system (EMRS) of hospital. Patients were followed up for 76.0 (36.0–236.0) days.

### Statistical Analyses

Continuous normal variables are presented as the mean ± standard deviation, and continuous abnormal variables are presented as the median (interquartile range, IQR). Categorical variables are presented as the number of cases (percentage). Continuous variables were compared by Student’s t-test or the Mann-Whitney *U* test, whereas categorical variables were compared using χ2 analyses or Fisher’s exact test. A *P*-value of < 0.05 (two-tailed) was considered significant. Data analyses were performed using SPSS version 26.0 (IBM, Armonk, NY, United States).

### Ethical Approval of the Study Protocol

This study protocol was performed in accordance with the 1975 Declaration of Helsinki and was approved by the Ethics Committee of the First Affiliated Hospital, College of Medicine, Zhejiang University (Hangzhou, China) (No. 2021-599). All data analyzed were anonymous.

## Results

### Patient Characteristics and Demographic Details

There were 73.8% (31/42) male patients and 26.2% (11/42) female patients. The mean age of patients was 50.1 ± 18.5 years old. A total of 45.2% (19/42) of the patients had predisposing diseases, including 9.5% (4/42) with chronic hematological disease, 4.8% (2/42) with hepatitis B virus (HBV) infection, 4.8% (2/42) with diabetes, 4.8% (2/42) with chronic kidney disease, 2.4% (1/42) with solid organ transplantation, 2.4% (1/42) with malignancy and 40.5% (17/42) with hypertension. In addition, 40.5% (17/42) of the patients had craniocerebral trauma, 26.2% (11/42) had hemorrhage in the CNS, 14.3% (6/42) had glioma and 4.8% (2/42) had craniocerebral abscess at admission. Overall, there were 71.4% (30/42) neurosurgical patients and 28.6% (12/42) non-neurosurgical patients. A total of 28.6% (12/42) of the patients had a history of stay in the intensive care unit (ICU). No difference in predisposing conditions was found between the neurosurgical and non-neurosurgical patients. The characteristics and demographic details are summarized in Table 1.

**TABLE 1:** The Clinical features of patients with *Candida* spp. in CSF.

**No.**	**Sex/**	**Symptoms**	**PDs**	**Neuro-**	**surgery**	**CSF**	**Deep**	**CSF**	**CSF**	**CSF)**	**Source**	**Candida.**	**Sensitivity** **(a/v/**	**Antifungal**	**Other**	**CSF**	**Probable** **Candida** **menigitis**	**outcome**
	**age**	**and**		**diseases**		**drainage**	**venous**	**glucose**	**protein**	**WBC**	**and times**	**spp**	**test** **(It/f)**	**treatment**	**source**	**other**		
		**sign**					**catheter**	**(mmol/L)**	**(g/L)**	**(× 10^6^/L)**	**of Positive CSF**				**of Candida**	**microbes**		
1	M/53	No	CKD, HT	glioma	No	No	Yes	1.6	5.1	90	Lumbar puncture × 1	C. catenulata		No	No	No	No	survival
2	F/19	fever	No	glioma	Tumour resection	LD; EVD	Yes	1.7	0.7	260	Lumbar puncture × 3	C. parapsilosis	S/S/S/S	Flu400mg × 7d→flu 800mg × 21d→ 400mg × 7d	No	No	Yes	survival
3	M/48	No	No	hemorrhage	ventricle drainage	LD; EVD	Yes	3.3	0.7	3	Lumbar puncture × 1	C. albicans	S/S/S/S	No	No	No	No	survival
4*	M/69	Mental change	HT	trauma	VPS; Lumbar cistern drainage	LD; VP	No	0.4	1.6	310	Catheter × 2	C. parapsilosis	S/S/S/S	Flu800mg × 3d	sputum	Staphylococcus capitis	Yes	death
5	M/68	headache	No	No	No	No	Yes	1.7	1.2	30	Lumbar puncture × 1	C. parapsilosis	S/S/I/S	(AmB + 5FC) × 14d→Flu 200mg*60d →→	No	Cryptococcus	No	survival
6	M/17	Mild fever	No	trauma	No	No	No	3.3	0.4	0	Lumbar puncture × 1	C. parapsilosis	S/S/S/S	No	No	No	No	survival
7*	M/74	Fever,	HT	hemorrhage	Haematoma clearance, craniotomy	LD; EVD	Yes	2.2	0.7	385	Catheter × 6	C. glabrata	S/I/S/I	Vori400mg × 14d→ 200mg × 16d	No	Klebsiella pneumoniae	Yes	survival
8*	F/63	No	No	hemorrhage	ventricle drainage	LD; EVD	Yes	4.3	0.7	0	Lumbar puncture × 1	C. albicans	S/S/S/S	No	No	Staphylococcus epidermidis	No	survival
9	M/39	fever	No	trauma	Haematoma clearance craniotomy	LD	Yes	1.1	0.8	370	Lumbar puncture × 1, Catheter × 4	C. glabrata	S/S/S/S	Flu200mg × 11d→ flu800mg ×b 25d	No	Staphylococcus epidermidis, Acinetobacter baumannii	Yes	survival
10	F/26	No	No	trauma/hemorrhage	Cranioplasty, VP, Lumbar cistern drainage	LP; VP	Yes	1.9	0.9	18	Catheter × 2	C. catenulata		No	No	Staphylococcus epidermidis,	No	survival
11	M/42	Fever, encephalocele	No	trauma	Haematoma clearance craniotomy	No	No	2.7	1.0	35	Lumbar puncture × 1	C. albicans	S/S/S/S	Vori400mg × 3d→Flu 400 × 14d→→	No	No	Yes	survival
12*	M/22	Fever,mental change	No	trauma	Haematoma clearance craniotomy	LD; EVD; VP	Yes	2.4	0.7	10	Catheter × 1	C. famata	S/R/R/R	Flu200md × 2d→ 600mg × 3d→200mg × 2d	No	Staphylococcus epidermidis, Acinetobacter baumannii	No	survival
13*	M/52	fever, mental change	No	trauma	CSF leakage repair; abscess clearance	LD; EVD	Yes	1.0	2.4	90	Lumbar puncture × 1 Catheter × 3	C. albicans		Flu400mg × 10d→ 200mg × 15d	sputum	No	Yes	survival
14	F/52	Mild fever	HBV, HT	glioma	Tumor resection	LD	Yes	4.5	5.6	700	Catheter × 1	C. albicans	S/S/S/S	No	No	Staphylococcus warneri; Acinetobacter baumannii	No	survival
15	M/57	no	HT	trauma	Haematoma clearance; craniotomy	EVD	No	2.9	0.9	3	Lumbar puncture × 1	C. albicans	S/S/S/S	Flu400mg × 3d→ 200mg × 7d	No	No	No	survival
16*	M/75	No	DM, HT	No	resection of vertebral tumor; CSF leakage repair	EVD	Yes	0.0	5.7	2800	Catheter × 1	C. albicans	S/S/S/S	Flu400mg × 11d→ Vori400mg × 16d	No	Klebsiella pneumoniae	No	death
17	F/51	Fever, headache	CKD, SOT HT	No	No	No	Yes	5.2	0.4	140	Lumbar puncture × 1	C. catenulata		(AmB + 5FC) × 14d→Flu 200mg*60d →→	No	Cryptococcus	No	survival
18	M/50	headache	HT	No	No	No	No	2.7	2.5	100	Lumbar puncture × 1	C. catenulata		No	No	Staphylococcus epidermidis,	No	survival
19	F/56	No	No	trauma hemorrhage	Haematoma clearance craniotomy	EVD	Yes	1.3	4.4	4800	Catheter × 1	C. parapsilosis	S/S/S/S	No	No	No	No	survival
20	M/71	fever	HT	trauma	Haematoma clearance	LD	Yes	2.3	1.4	20	Catheter × 5	C. albicans	S/S/S/S	Flu400mg × 5d→ Cease treatment	sputum	Staphylococcus epidermidis,	Yes	death
21*	F/52	headache	HT	trauma	Haematoma clearance, CSF leakage repair; craniotomy	LD	Yes	3.1	1.9	470	Catheter × 1	C. glabrata	S/S/R/I	Flu600mg × 7d→ vori400mg × 1d	**No**	Klebsiella pneumoniae	No	survival
22	M/82	No	DM		No	No	Yes	8.0	4.8	45	Lumbar puncture × 1	C. parapsilosis		No	No	No	No	death
23	F/62	fever	HT	hemorrhage	VPS; ventricle drainage	LD; EVD;VP	Yes	3.8	0.4	3	Catheter × 1	C. parapsilosis		Cease treatment	No	Staphylococcus haemolyticus	Yes	death
24	M/7	fever		trauma hemorrhage	CSF leakage repair	LD;VP	Yes	3.4	0.5	280	Catheter × 2	C. albicans	S/S/S/S	Flu400mg × 24d	No	No	Yes	survival
25	M/36	seizure	HBV	glioma	CSF leakage repair	LD	No	2.1	1.2	120	Lumbar puncture × 1	C.tropicalis	S/I/R/R	No	No	No	No	survival
26	M/72	Fever, headache	HT	glioma	Tumour resection CSF leakage repair	LD;EVD	Yes	2.5	1.2	200	Lumbar puncture × 2	C. parapsilosis	S/S/S/S	Flu400mg × 11d→ 200mg × →	blood	No	Yes	survival
27	M/32	fever		trauma	No	No	No	3.3	0.3	42	Lumbar puncture × 1	C. catenulata		No	No	No	No	survival
28	M/62	Fever, mental change	No	No	No	No	No	3.8	1.1	90	Lumbar puncture × 1	C. parapsilosis	S/S/S/S	Flu400mg × 3d	No	No	No	survival
29	M/65	Fever, headache	No	No	No	No	No	4.6	1.1	20	Lumbar puncture × 1	C. parapsilosis	S/S/S/S	No	No	No	No	survival
30	F/58		malig nancy		No	No	No	4.6	4.8	50	Lumbar puncture × 1	C. albicans		No	No	No	No	survival
31	M/22	fever	No	trauma	Haematoma clearance craniotomy	LD	Yes	1.4	1.1	88	Lumbar puncture × 1 Catheter × 2	C. glabrata	S/R/R/R	No	blood	Staphylococcus epidermidis,	Yes	death
32	M/46	Fever	No	No	No	No	No	3.7	0.4	28	Lumbar puncture × 1	C. parapsilosis	S/S/S/S	No	No	Staphylococcus kloosii,	No	survival
33*	M/71	No	No	hemorrhage	Haematoma clearance	LD	Yes	2.2	2.1	30	Catheter × 1	C.tropicalis	S/R/R/R	Flu400mg × 3d	No	No	No	survival
34	M/18	encephalocele	No	trauma	Haematoma clearance; craniotomy	LD; EVD; VP	Yes	2.3	1.0	1	Catheter × 1	C. albicans		Flu200mg × 9d	sputum	Staphylococcus epidermidis, Acinetobacter baumannii; Klebsiella pneumoniae	No	survival
35*	M/49	Coma	HBV HT	trauma hemorrhage	Haematoma clearance, abscess clearance	LD; EVD; VP	Yes	3.6	0.5	10	Catheter × 6	C. parapsilosis	S/S/S/S	Flu200mg × 18d→ Vori400mg × 37d→ 200mg × 7d→flu 400mg × 10d	No	Staphylococcus auris	Yes	survival
36*	M/59	fever	No	abscess	CSF leakage repair; abscess clearance; Haematoma clearance; craniotomy	LD; EVD; VP	Yes	4.9	1.6	100	Lumbar puncture × 1	C.tropicalis	S/R/I/R	Flu400mg × 10d 200mg × 28d	No	No	Yes	death
37	M/51	Fever,Coma	HT	hemorrhage	external ventricular drainage; VPS	LD; EVD; VP	Yes	0.8	2.4	980	Catheter × 3	C. catenulata		Flu800mg × 2d→ 400mg × 9d	No	Enterobacter cloacae	Yes	death
38	F/66	No	HT	glioma	Tumour resection	EVD	No	-	-	30000	Catheter × 1	C. parapsilosis	S/S/S/S	No	No	No	No	survival
39	M/26	Fever, headache	No	hydro cephalus	external ventricular drainage,	EVD	No	2.5	2.4	230	Lumbar puncture × 1	others	S/S/S/S	Delay:Vori400 × 6d→ cease treatment	No	No	Yes	death
40*	M/63	fever	HT	hemorrhage	Haematoma clearance	VPS; EVD; VP	Yes	5.4	1.4	0	Catheter × 1	C. parapsilosis	S/S/S/S	Flu800mg × 30day	No	No	Yes	death
41*	F/47	fever	No	trauma	Haematoma clearance; abscess clearence	LD; EVD; VP	Yes	0.05	0.6	10	Catheter × 5	C. albicans	S/S/S/S	Flu600mg × 10d→ 200mg × 7d	sputum	Pseudomonas aeruginosa; Corynebacterium striatum	Yes	death
42#	M/55	Fever, mental change	HT	No	No	No	No	5.5	2.9	180	Lumbar puncture × 1	C. parapsilosis	S/S/S/S	Flu800mg × 3d→vori 400mg × 1d→ cease treatment	**No**	No	Yes	death

M, male; F, female; PDs, predisposing diseases; HT, hypertension; CKD, chronic kidney disease; SOT, solid organ transplantation; LD, lumbar cistern drainage; EVD, ventricular drainage; VP, ventricular peritoneal shunt; f, fluconazole; v, voriconazole; a, amphotericin B deoxycholate; It, itroconazole; S, sensitivity; I, intermediate; R, resistance; →, more than 2 months. *, patients in intensive care unit; #, DNA sequence of *C. parapsilosis* was also found in CSF.

### Isolation of *Candida* spp. From CSF Samples

*Candida albicans* (*C. albicans*) was isolated from 28.6% (12/42) of the patients, *Candida parapsilosis* (*C. parapsilosis*) from 35.7% (15/42), *Candida glabrata* (*C. glabrata*) from 9.5% (4/42), *Candida tropicalis* (*C. tropicalis*) from 7.1% (3/42), and other species from 19.0% (8/42). The percentage of *C. albicans* cases was 36.7% (11/30) and that of non-*C. albicans* cases was 63.3% (19/30) among the neurosurgical patients; meanwhile, the percentage of *C. albicans* cases was 8.3% (1/12) and that of non-*C. albicans* cases was 91.7% (11/12) in the non-neurosurgical patients (*P* = 0.128). Twenty-nine patients had 1 episode of *Candida* spp., 3 patients had 2 episodes of *Candida* spp., and 10 patients had ≥ 3 *Candida* spp. isolates. Overall, 56.7% (17/30) of cases had a single episode of *Candida* spp., and 43.3% (13/30) of cases had repeated episodes of *Candida* spp. among patients with neurosurgery, whereas 100% (12/12) of cases had a single episode of *Candida* spp. from CSF among non-neurosurgical patients (*P* = 0.006). In addition, positive culture and DNA sequencing of *C. parapsilosis* by next-generation sequencing (NGS) were also found in CSF in one patient (Patient 42).

There were 3 patients (9, 13, and 31) with repeated positive CSF samples both from lumbar puncture and drain devices, 19 with single positive CSF samples from lumbar puncture, 2 (2 and 26) with repeated positive CSF samples from lumbar puncture, 10 with single positive CSF samples from indwelling drain devices and 8 patients (4, 7, 10, 20, 24, 35, 37, and 41) with repeated positive CSF samples from indwelling drain devices.

Patients in the ICU had a higher positive rate of CSF samples from drain devices [83.3% (10/12) vs. 36.7% (11/30), *P* = 0.019] but a lower positive rate of CSF samples from lumbar puncture [25.0% (3/12) vs. 70.0% (21/30), *P* = 0.008] than those not in the ICU.

### Diagnosis of Probable *Candida meningitis*

Five patients (4, 13, 20, 34, and 41) had positive sputum cultures, and 2 patients (26 and 31) had positive blood cultures. Eighteen of 42 (42.9%) patients were considered to have PCM, including one non-neurosurgical patient (42) and 17 neurosurgical patients. Of those neurosurgical patients with PCM, 17.6% (3/17) of the patients (9, 13, and 31) had positive CSF samples obtained both from lumbar puncture and indwelling drain devices, 29.4% (5/17) had positive CSF samples obtained from lumbar puncture, and 52.9% (9/17) had positive CSF samples obtained from indwelling drain devices. Furthermore, among those 8 neurosurgical PCM patients with positive lumbar puncture samples, 2/8 (25.0%) patients had repeated positive CSF samples, and 6/8 (75.0%) had a single positive CSF sample. Meanwhile, among the 12 neurosurgical PCM patients with positive CSF samples obtained from indwelling drainage devices, 10/12 (83.3%) patients had repeated positive CSF samples, and 2/12 (16.7%) had a single positive sample.

In total, 24 patients had positive cultures of CSF from lumbar puncture (22 single positive cultures + 2 repeated positive cultures), and 21 patients had positive cultures from indwelling drainage samples (10 single positive cultures + 11 repeated positive cultures). Single positive cultures from lumbar puncture had 77.8% (95% CI: 40.2–96.1%) sensitivity and 0–25.3% specificity, whereas repeated positive samples from lumbar puncture had 22.2% (95% CI: 4.0–59.8%) sensitivity and 100.0% specificity for the diagnosis of PCM. A single positive CSF culture from drainage devices had a sensitivity of 16.7% (95% CI: 2.9–49.1%) and a specificity of 11.1% (95% CI: 0.6–49.3%), whereas a repeated positive culture from drainage devices had a sensitivity of 83.3% (95% CI: 50.9–97.1%) and a specificity of 88.9% (95% CI: 50.6–99.4%). Altogether, single positive CSF cultures had a sensitivity of 7.7% (95% CI: 0.4–37.9%) and a specificity of 20.7% (95% CI: 8.7–40.3%). Repeated CSF samples from the same or different routines had 66.7% (95% CI: 41.2–85.6%) sensitivity and 95.8% (95% CI: 76.9–99.8%) specificity for the diagnosis of PCM.

Furthermore, bacteria were isolated from CSF in 16/30 (53.3%) neurosurgical patients, including *Staphylococcus* spp. from 12/30 (40/0%) patients, *Klebsiella pneumoniae* from 4/30 (13.3%) patients, *Acinetobacter baumannii* from 4/30 (13.3%) patients, *Enterobacter cloacae* from 1/30 (3.3%) patient, *Pseudomonas aeruginosa* from 1/30 (3.3%) patient and *Corynebacterium striatum* from 1/30 (3.3%) patient. The CSF isolation rates of bacteria were 52.9% (9/17) in PCM and 53.8% (7/13) in non-PCM patients among those who underwent neurosurgery (*P* = 0.961).

We found that neurosurgery [OR: 14.4 (1.6–126.1), *P* = 0.004], lumbar drainage [OR: 5.8 (1.5–23.3), *P* = 0.009], VP shunt [(OR: 5.6 (1.2–25.8), *P* = 0.020)], external ventricular drainage [(OR: 4.7 (1.3–17.7), *P* = 0.018], CRP ≥ 10.0 mg/L [(OR: 4.9 (1.3–18.1), *P* = 0.034] and postsurgical broad-spectrum antibiotics [OR: 9.5 (1.8–50.5), *P* = 0.004] were associated with PCM. Deep vein catheterization [(OR: 2.5 (0.6–9.9), *P* = 0.186] and ICU stay [OR: 2.4 (0.6–9.5), *P* = 0.200] were marginally associated with PCM. However, sex, age, predisposing disease, hypoalbumin (< 35 g/L), anemia (< 110 g/L) and cerebral hemorrhage were not associated with PCM.

The CSF protein level of 1.2 (1.0–2.4) g/L for patients with PCM was close to the protein level of 1.1 (0.4–4.8) for patients without PCM (*P* = 0.636), and the CSF WBC level of 100.0 (61.5–215.0) × 10^6^ cells/L in patients with PCM was slightly higher than that of 42.0 (3.0–100.0) × 10^6^ cells/L for patients without PCM (*P* = 0.347). CSF glucose was 2.6 ± 1.6 mmol/L in patients with PCM and 3.2 ± 1.6 mmol/L in patients without PCM (*P* = 0.248). The ICP was 180.0 (105.0–310) mmH_2_O in PCM patients and 120.0 (80.0–135.0) mmH_2_O in non-PCM patients (*P* = 0.073).

### CT and/or MRI Image Findings

CT and/or MRI images were available in 76.2% (32/42) of patients, including 13 PCM patients and 19 non-PCM patients. Of those 13 patients with PCM, 8/13 (61.5%) had enlarged ventricles, 4/13 (30.8%) had parenchymal hemorrhage, 4/13 (30.8%) had hydrocephalus, 1/13 (7.7%) had abnormal basal ganglia changes, 2/13 (15.4%) had abscess changes, 1/13 (7.7%) had parenchymal softening, and 1/13 (7.7%) had enhanced meningeal ganglia. PCM patients had a trend of a higher incidence of enlarged ventricles than non-PCM patients [61.5% (8/13) vs. 36.8% (7/19), *P* = 0.169]. No other significant difference was found in CT and/or MRI imaging findings between the groups (Table 2).

**TABLE 2:** Comparisons of the radiology of patients with and without PCM.

**Radiology**	**PCM (*n* = 13) [*n* (%)]**	**Non-PCM (*n* = 19) [*n* (%)]**	***P* value**
Dilated brain ventricle	8 (61.5)	7 (36.8)	0.169
Cerebral hemorrhage	4 (30.8)	4 (21.1)	0.684
Hydrocephalus	4 (30.8)	2 (10.5)	0.194
Basal ganglia changes	1 (7.7)	3 (15.8)	–
Abscess	2 (15.4)	1 (5.3)	–
Parenchymal softening	1 (7.7)	1 (5.3)	–
Enhanced meningeal	1 (7.7)	0	–

*PCM, probable Candida meningitis.*

### Treatment and Outcome

Of the 42 patients, 26 received antifungal treatment, including 2 patients initially treated with AmB + 5FC (0.5–0.7 mg/kg/d), 3 initially treated with voriconazole (200 mg iv bid), 18 initially treated with fluconazole (200–800 mg/d) as induction treatment and 4 initially treated with fluconazole and then switched to voriconazole (Table 1). Two patients (5 and 17) who received antifungal treatment were eventually confirmed to have cryptococcal meningitis, although *Candida* spp. were initially isolated in the CSF samples. In particular, 16/18 (88.9%) PCM patients (including two patients (20 and 42) who ceased treatment) and 10/24 (41.7%) patients without PCM were treated with antifungal regimens. There were 31 patients with antifungal susceptibility testing data available. Our data showed that non-*C. albicans* cases exhibited a higher rate of intermediate/resistance to azoles than *C. albicans* [36.4% (8/22) vs. 0 (0/9), *P* = 0.068] (Tables 1, 3). Ten patients died during the follow-up. Of those, the hospitalized mortality rates were 50.0% (9/18) in PCM patients and 4.8% (1/24) in non-PCM patients. The odds ratio of mortality was 23.0 (2.5–208.6) for PCM patients compared with non-PCM patients (*P* = 0.001).

**TABLE 3:** Number of *Candida* isolates for intermediate/resistance to antifungal agents tested.

***Candida species***	**Amphotericin B**	**Vori conazole**	**Itra conazole**	**Flu conazole**	**5-flucytosine**
*C. albicans* (*n* = 9)	0	0	0	0	0
*C. parapsilosis* (*n* = 13)	0	0	0	0	0
*C. glabrata* (*n* = 4)	0	2	2	1	0
*C. tropicalis* (*n* = 3)	0	3	3	3	0
Other species (*n* = 2)	0	1	1	1	1

## Discussion

*Candida meningitis* is a rare but emerging problem in clinical practice. A previous study suggested that the occurrence of *C. meningitis* was associated with low body weight in infants and neurosurgery in adults (Marr et al., 1994; Nguyen and Yu, 1995; Geers and Gordon, 1999). However, the diagnostic criteria and clinical characteristics of *C. meningitis* remain unclear due to the rarity of the disease. In the present study, we found that (1) non-*C. albicans* was responsible for nearly 70% of *Candida* episodes; (2) neurosurgery, drainage devices, high CRP levels and usage of broad-spectrum antibiotics after surgery were associated with PCM; (3) although a single positive CSF sample drawn through an indwelling device was difficult to assess, repeated episodes of positive CSF samples displayed high specificity and low sensitivity for the diagnosis of PCM; and (4) episodes of bacteria in CSF and the hospitalized mortality of PCM patients were both 50.0%.

Some predisposing conditions, such as long-term steroid usage, chemotherapy, and intravenous catheters, are associated with PCM. Neurosurgery is also recognized as a risk factor for Candida meningitis, especially shunt devices, which are closely related to CSF *Candida* infection (Montero et al., 2000; Bridges et al., 2017; Chen et al., 2020). In the present study, we found that approximately 70% of patients had a history of neurosurgery, and 30% did not. Neurosurgery and drainage devices were associated with PCM. The possible reason for *Candida* infection caused by neurosurgery and drainage devices might be related to the disruption of the blood-brain barrier. External ventricular and lumbar drainage devices both contribute to ventriculitis (Scheithauer et al., 2010; Citerio et al., 2015). However, the different impacts of external ventricular drainage and lumbar drainage on CNS infection are debatable. One study found evidence that lumbar drainage was associated with a low rate of ventriculitis (Schade et al., 2005), but another study indicated that lumbar drainage was a significant risk factor for ventriculitis (Scheithauer et al., 2010). CSF collected by lumbar puncture had a higher diagnostic accuracy than that collected by ventriculostomy for the diagnosis of CNS infection (Finger et al., 2020). Drainage device infection usually occurs after the surgical procedure and is believed to result from contamination (Lipton et al., 1984). Our study also indicated that VP shunts, lumbar drainage devices, and external ventricular drainage were risk factors for *C. meningitis*, and we speculated that contamination more easily occurred in drainage devices.

Twelve patients without neurosurgery had episodes of *Candida* in the CSF sample. Some studies indicated that patients with extracranial factors (such as abdominal surgery, recent broad-spectrum antibiotic therapy, indwelling catheters, malignancy and steroid use) were at high risk for the occurrence of *C. meningitis* (Bridges et al., 2017). In our study, we found that 50.0% (6/12) of patients without neurosurgery had predisposing diseases, but only one patient without neurosurgery had PCM. These data suggested that PCM in non-neurosurgical patients was rare, and the majority of episodes of *Candida* in CSF were from contamination during lumbar puncture in non-neurosurgical patients. In addition, we found that neurosurgery and drainage devices, rather than predisposing diseases, were risk factors for the development of PCM, revealing that the risk factors for the development of PCM in neurosurgical patients were different from those in non-neurosurgical patients.

Some studies suggested that broad-spectrum antibiotic therapy was a contributor to PCM, and 50% of patients suffered from antecedent bacterial meningitis (Nguyen and Yu, 1995). In fact, previous studies indicated that infection with *Candida* spp. was always associated with concurrent bacterial infection (Peres-Bota et al., 2004; Tarumoto et al., 2014). In the present study, 52.9% of PCM patients had CSF bacterial infection, which was close to the incidence of 53.8% in non-PCM patients, indicating that broad-spectrum antibiotics were a concurrent treatment for coinfection rather than a cause of CNS *Candida* episodes. Thus, it was reasonable that a high CRP level was associated with the incidence of PCM.

Some data indicated diffuse or multiple miliary nodules and hyperintense signals on DWI, while non-significant signal changes in T1WI and T2WI on MRI images were found among preterm infants with *C. albicans* infection (Mao et al., 2012). Marked hydrocephalus and leptomeningeal enhancement were observed on brain MRI of patients with *Candida dubliniensis* infection (Yamahiro et al., 2016). Our data suggested that enlarged ventricles, parenchymal hemorrhage and hydrocephalus were the most common findings in PCM patients. However, no significant difference was found in enlarged ventricles (*P* = 0.169), parenchymal hemorrhage (*P* = 0.684) or hydrocephalus (*P* = 0.150) between patients with and without CNS *Candida* infection (Table 2). It was previously noted that patients with postneurosurgical *C. meningitis* usually had a recent history of bacterial meningitis, antibiotic therapy and multiple surgeries involving the CNS (Lipton et al., 1984). PCM patients had a trend of higher ventricle involvement in our study (61.5% vs. 36.8%, *P* = 0.169), which was thought to be associated with CNS infection through indwelling devices (Scheithauer et al., 2010; Citerio et al., 2015; Finger et al., 2020). We hypothesize that the limited sample size, coinfection of bacteria and surgical comorbidities might make it difficult to outline the unique radiological characteristics of PCM patients in our study.

Although all patients had episodes of *Candida* in the CSF, only 40% of patients were diagnosed with PCM. Furthermore, 41.6% of patients without PCM and 88.9% of patients with PCM were treated with antifungal regimens. A previous study also revealed that all patients with CSF *Candida* episodes accepted antifungal treatment, although only 50% of patients were considered PCM patients (Geers and Gordon, 1999). Therefore, it is difficult to determine true PCM patients in clinical practice. These data suggest that the diagnostic strategy of PCM should be revised urgently. Although a single episode of *Candida* spp. in CSF was insufficient for the diagnosis of PCM, repeated episodes of *Candida* spp. had approximately 66.7% sensitivity and 95.8% specificity for the diagnosis of PCM. These results indicated that the diagnosis of PCM by single or repeated positive CSF samples drawn through lumbar puncture or indwelling devices is insufficient for diagnostic performance. Notably, repeated positive samples from lumbar puncture had only ∼11.7% sensitivity and 100.0% (71.7–100.0%) specificity for the diagnosis of PCM. Another important issue is that most neurosurgical PCM patients in our study did not have host risk factors such as neutropenia, malignancy and immunosuppressive therapy, demonstrating that the risk factors for postneurosurgical patients with *C. meningitis* are divergent from those for non-neurosurgical patients. Thus, a novel strategy for the diagnosis of CM is needed. Some studies revealed that CSF (1,3)-β-D-glucan was a surrogate marker for PCM, and the persistence of (1,3)-β-D-glucan in CSF was associated with the clinical and microbiological failure of PCM (Lyons et al., 2015; Ceccarelli et al., 2016; Farrugia et al., 2016). Although elevated CSF (1,3)-β-D-glucan was not specifically present in CNS infections of *Aspergillus*, *Cryptococcus* and *Histoplasma* (Rhein et al., 2014; Chen et al., 2017; Myint et al., 2018), CSF (1,3)-β-D-glucan testing may still be a useful surrogate marker of *C. meningitis* (Davis et al., 2020). Although it seemed that a single episode of CSF *Candida* was insufficient for PCM diagnosis, a study indicated that normal CSF parameters were found in 43% (3/7) of infants with *C. meningitis*, and only 37% (7/19) of them had positive blood cultures for *Candida* (Cohen-Wolkowiez et al., 2007). Some CSF samples of PCM often resemble bacterial profiles, and cultures can be falsely negative (Bridges et al., 2017). Even culture-negative *C. meningitis* diagnosed by the detection of *Candida* mannan antigen in CSF was reported (Biesbroek et al., 2013). Thus, CSF culture combined with CSF (1,3)-β-D-glucan assay should be considered in patients who are suspected of infectious meningitis of unknown cause. Alternatively, quantitative *Candida* colonies were used to assist in the diagnosis of candidiasis (Pfeiffer et al., 2011; Zhou et al., 2017). It is unknown whether quantitative analysis of *Candida* colonies in CSF obtained from lumbar puncture or indwelling devices is helpful for the diagnosis of PCM.

Previous studies indicated that non-*C. albicans* species accounted for over two-thirds of isolates from blood in China (Xiao et al., 2020; Zhang et al., 2020). In the present study, we found that the percentage of non-*C. albicans* exceeded 70%, and non-*C. albicans* species had higher trends of resistance to azoles than *C. albicans*. Our data depicted a trend of potential risk for treatment failure of PCM, a high rate of misdiagnosis in patients with CSF episodes of *Candida* and high mortality in PCM patients. Importantly, the mortality of PCM was 50.0% in our study, which was consistent with previous reports of 42–50% mortality (Geers and Gordon, 1999; Chaussade et al., 2021). These results suggested that antifungal agents should be initiated promptly in patients whose cerebrospinal fluid is positive for *Candida* and who have one or more of the identified risk factors.

There were some limitations in our study. First, we did not perform a CSF (1,3)-β-D-glucan assay to further support our diagnosis. Second, our data are based on a small sample size, and we were unable to identify the risk factors associated with mortality. Third, our study is a retrospective study, which is prone to certain biases.

In summary, our study showed that neurosurgery, indwelling drainage devices and usage of broad-spectrum antibiotics after neurosurgery led to a high risk for *Candida* CNS infection. Although it was difficult to diagnose CNS *Candida* infection from a single episode of *Candida* spp. in the CSF, repeated episodes of *Candida* spp. in the CSF had low sensitivity for diagnosis of PCM. CSF (1,3)-β-D-glucan, *Candida* colony quantitation and next-generation sequencing might be considered helpful for the improved diagnosis of CNS *Candida* infection.

## Data Availability Statement

The original contributions presented in the study are included in the article/supplementary material, further inquiries can be directed to the corresponding authors.

## Ethics Statement

This study protocol was performed in accordance with the 1975 Declaration of Helsinki and was approved by the Ethics Committee of the First Affiliated Hospital, College of Medicine, Zhejiang University (Hangzhou, China) (No. 2021-599). All data analyzed were anonymous. The ethics committee waived the requirement of written informed consent for participation.

## Author Contributions

LX designed the study, drafted the manuscript, and analyzed and interpreted the data. HZ performed the study and performed the follow-ups. MZ and GL collected the data. HL reread the radiological images. All authors reviewed and approved the final manuscript.

## Conflict of Interest

The authors declare that the research was conducted in the absence of any commercial or financial relationships that could be construed as a potential conflict of interest.

## Publisher’s Note

All claims expressed in this article are solely those of the authors and do not necessarily represent those of their affiliated organizations, or those of the publisher, the editors and the reviewers. Any product that may be evaluated in this article, or claim that may be made by its manufacturer, is not guaranteed or endorsed by the publisher.
